# Novel sulphonic acid liquid crystal derivatives: experimental, computational and optoelectrical characterizations†

**DOI:** 10.1039/d1ra02517a

**Published:** 2021-08-17

**Authors:** Latifah A. Alshabanah, Laila A. Al-Mutabagani, Sobhi M. Gomha, Hoda A. Ahmed, Saheed A. Popoola, Mohamed Shaban

**Affiliations:** Department of Chemistry, College of Science, Princess Nourah Bint Abdulrahman University Riyadh 11671 Saudi Arabia; Department of Chemistry, Faculty of Science, Cairo University Cairo 12613 Egypt ahoda@sci.cu.edu.eg; Chemistry Department, Faculty of Science, Islamic University of Madinah Al-Madinah Al-Munawwarah 42351 Saudi Arabia smgomha@iu.edu.sa; Chemistry Department, College of Sciences, Yanbu, Taibah University Yanbu 30799 Saudi Arabia; Department of Physics, Faculty of Science, Islamic University in Almadinah Almonawara Almadinah 42351 Saudi Arabia; Nanophotonics and Applications Labs, Department of Physics, Faculty of Science, Beni-Suef University Beni-Suef 62514 Egypt

## Abstract

A novel liquid crystal homologous series based on the benzene sulphonic acid moiety, namely (*E*)-4-((4-((4-(alkoxy)benzoyl)oxy)benzylidene)amino)benzenesulfonic acid (Sn), was synthesized and examined *via* different experimental and theoretical measurements. The four synthesized members have terminally connected alkoxy chain groups, which vary between 6 and 12 carbons. FT-IR and NMR spectroscopy, as well as elemental analyses, were used to confirm their molecular structures. Mesomorphic and optical investigations of the prepared homologues were also conducted using differential scanning calorimetry (DSC) and polarized optical microscopy (POM). The DSC and POM characterization revealed that all of the synthesized sulphonic acid members are monomorphic, exhibiting a pure smectic A (SmA) mesophase with enantiotropic properties. Moreover, all compounds in the group have high thermal transition temperatures. The terminal electron-withdrawing group –SO_3_H plays a considerable role in the stabilization of the molecule, which in return resulted in high thermal SmA stability. Furthermore, the experimental data relating to the mesophase behavior were substantiated *via* computational studies using the DFT approach. In addition, the terminal –SO_3_H moiety has an essential impact on the thermal and physical parameters of possible geometries. All members of the synthesized Sn series exhibit ohmic behavior with electrical resistance in the GΩ range, as revealed by electrical measurements. The S10 electrode had the highest electrical conductivity: 35.16 pS. It also showed two direct optical band gaps of 3.58 and 3.23 eV with Urbach energies of 1261.1 and 502.4 meV. Upon decreasing the number of carbon atoms to *n* = 6, the main bandgap for S6 reduced to 3.3 eV. The highest conductivity, good absorption, and two large bandgaps recorded for the chain derivative S10 make it suitable for investigations relating to energy-based applications.

## Abstract 2

A novel liquid crystal homologous series based on the benzene sulphonic acid moiety, namely (*E*)-4-((4-((4-(alkoxy)benzoyl)oxy)benzylidene)amino)benzenesulfonic acid (Sn), was synthesized and examined *via* different experimental and theoretical measurements.

## Graphical abstract

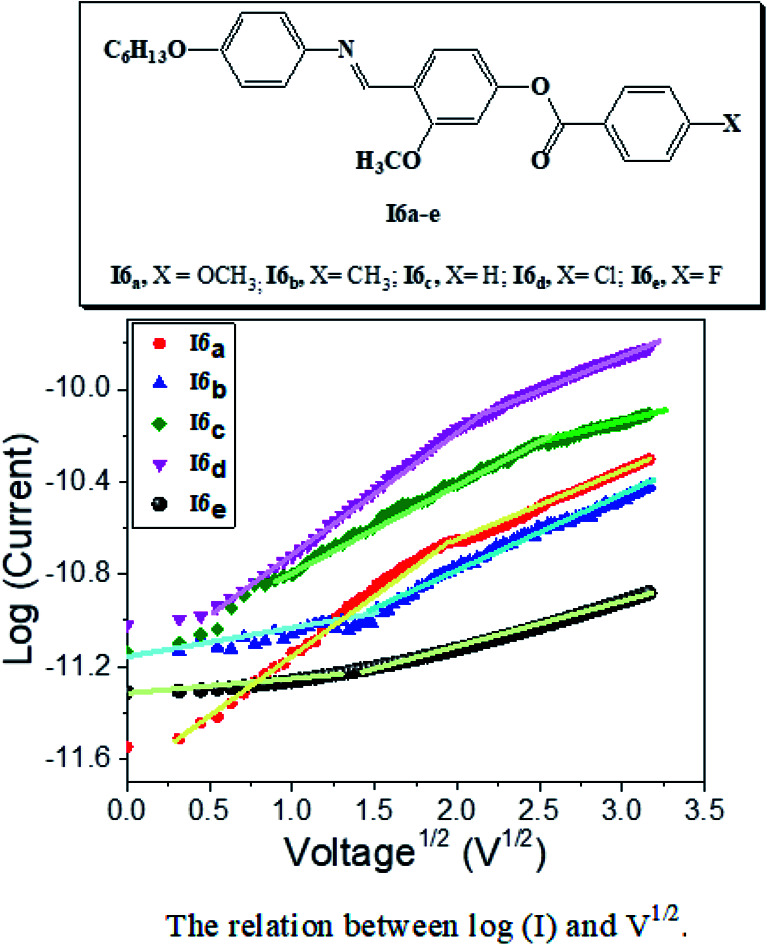

## 1. Introduction

Nowadays, organic solar cells are highly promising and cost-effective compared to traditional cells. As such, a large number of research studies on the application of organic compounds as photosensitizers for solar cells have been documented.^1–4^ Moreover, low molar mass molecule solar cells possess great potential.^5–9^ Innovative properties of organic solar cells, such as light-weight, flexibility, low cost, and solution processability, have attracted considerable attention from researchers and technological engineering. Furthermore, the modern organic solar cell types have proven to be commercially inexpensive with excellent efficiencies.^10^ For solar energy applications, such as catalytic photo-degradation of dyes, solar hydrogen generation, photo-electrochemical water splitting, and solar cells, bandgap engineering and optical property control are critical parameters of our interest.^11–15^

Recently, mesomorphic materials have been proven to have broad technological applications as light emitting diodes, displays and photoconductors.^16–18^ The impact of symmetrical cells filled with liquid crystal (LC) compounds has been previously investigated.^19–21^ In order to enhance the ion conductivity of engineered materials, they should be incorporated with a liquid crystalline derivative.^22^ The smectic ordering of liquid crystals, including ion-conductive tails, results in phases with alternating ion conductive/insulating layers.^23,24^ Moreover, the mesogen cores increase the ion-conductive tails between the insulating parts, thereby influencing in-plane ion transport. Similar behaviors of anisotropic character have been documented within LC polymer mesophases.^23,25^

The design of new structural shapes to achieve the desired applicable properties in industry is one of our interests.^26–30^ Thus, the selection of the terminal tail, the terminal and lateral polar substituents, and the mesogenic cores are important criteria in developing novel mesomorphic materials for proper characteristic technological applications. In addition, the molecular geometry enables some considerable modifications in the mesophase properties and plays an important role in the formation, kind, and thermal mesomorphic stability of the reported mesophases.^26–30^

Liquid crystalline systems with a terminal acid moiety have the ability to form strong supramolecular interactions.^31–34^ There is a report of LCs formed through the interaction of aromatic carboxylic acids.^35^ In addition, several types of materials have been formed by the interaction of complementary molecules with their LC behavior being crucially dependent on the shape of the resulting supramolecular systems. Generally, dimerization links connecting two molecular species^36,37^ have been proven to be optically anisotropic, which agrees with the main properties of liquid crystal molecules.

On the other hand, the inclusion of electron terminal polar functional groups in the LC skeleton may strongly influence their polarizability and/or polarity, as well as their geometric structures. Consequently, this affects the transition phase temperature, kind of mesophase and other physical and geometrical parameters essential for better characteristics of the LC materials.^38–41^

The Schiff base moiety maintains the rigidity as well as the linearity of the molecular geometry, which enhances the mesomorphic stability. More reports of low molecular mass Schiff base systems and twist bend nematic mesophases have been investigated.^42–44^ On the other hand, the conjugative interactions between the –COO- group and the phenyl rings play an important role in getting better mesomorphic behaviour. Moreover, the terminal flexible chains or polar compact substituents have essential roles in the phase transition properties.^45^

The goal of our present work is to synthesize new azomethine derivatives of a terminal sulphanilic acid moiety, with changeable terminal alkoxy chain length, namely (*E*)-4-((4-((4-(alkoxy)benzoyl)oxy)benzylidene)amino)benzenesulfonic acid, Sn. This study aims to investigate the mesophase properties of the prepared homologues series *via* experimental and theoretical approaches. Additionally, we intend to evaluate the effect of the different terminal length of the attached alkoxy chain on their mesomorphic properties. To achieve our objectives, electric and optical property, electric resistance, conductance, energy gap and Urbach energy measurements were conducted on the synthesized new azomethine derivatives. Furthermore, a computational approach was employed to corroborate the experimental data.
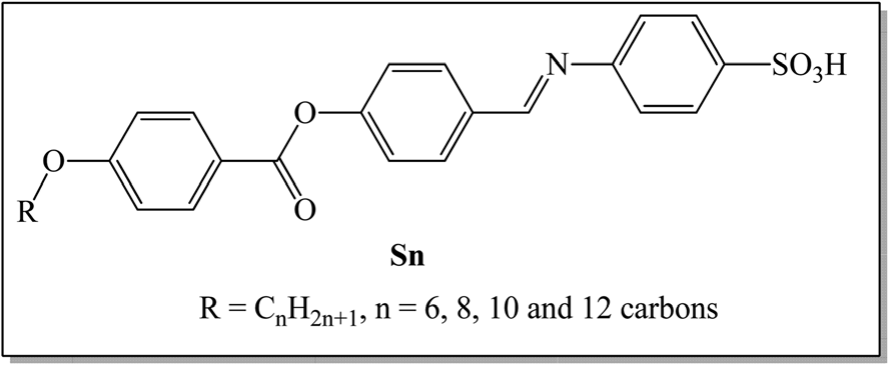


## 2. Experimental

### 2.1. Synthesis

**Scheme 1:**
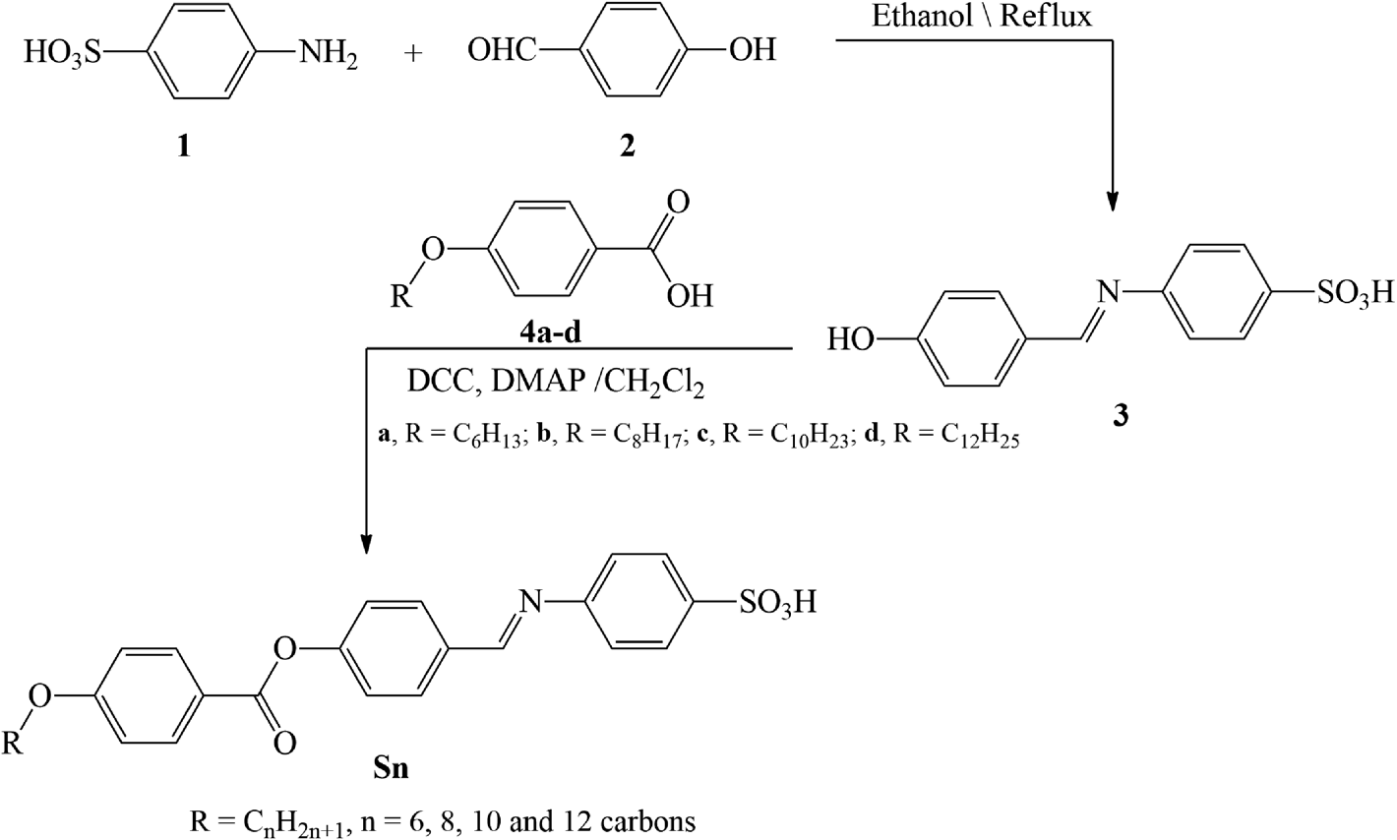
Synthesis route to the title Sn compounds.

Hydrazones and Schiff bases are important precursors in the synthesis of various valuable organic compounds that are employed in a variety of applications.^46–52^ The present homologue Sn was prepared according to the following scheme (Scheme 1):

#### 2.1.1. Synthesis of (*E*)-4-((4-hydroxybenzylidene)amino)benzenesulfonic acid (3)

Yellow solid, m.p. = 312–314 °C, (Lit m.p. > 300 °C).^53^

#### 2.1.2. Synthesis of (*E*)-4-((4-((4-(alkoxy)benzoyl)oxy)benzylidene)amino) benzenesulfonic acid, Sn

**Fig. 1:**
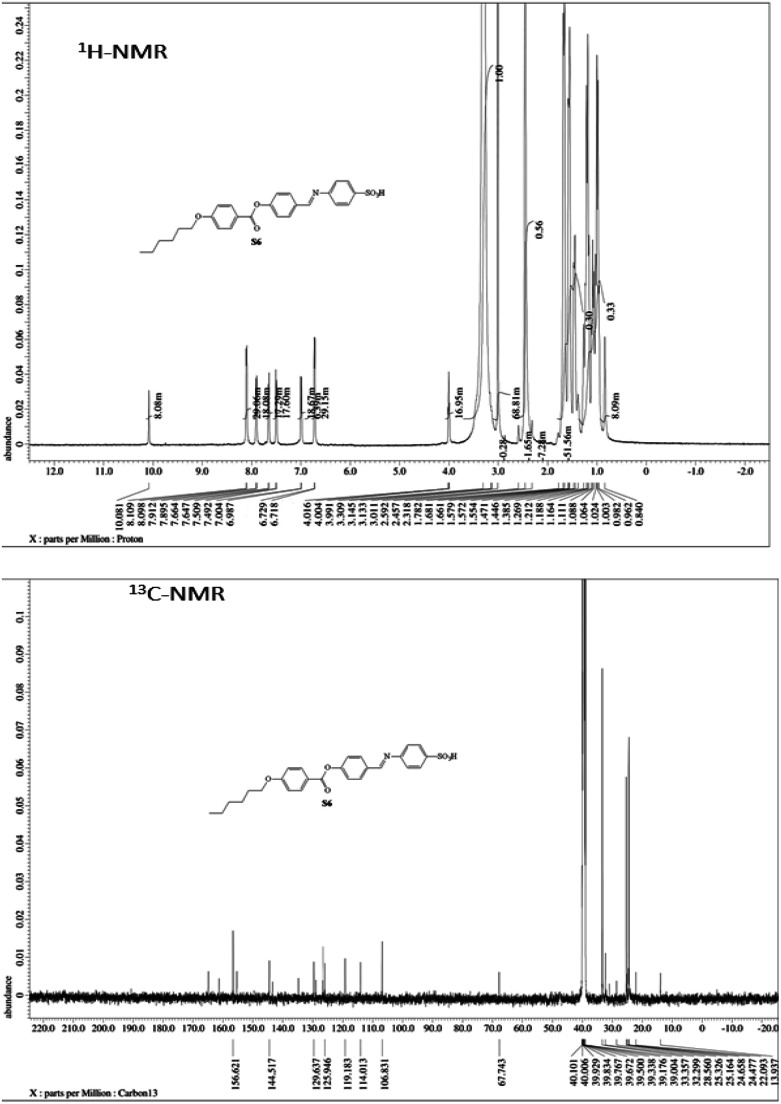
^1^H-NMR and ^13^C-NMR spectra of compound S6.

The method is provided in the ESI† with all of the physical data of the products Sn. The ^1^H-NMR and ^13^C-NMR spectra of derivative S6 are depicted in Fig. 1, as a representative example.

### 2.2. Computational details

All of the compounds studied were fully optimized without geometrical restriction using the GAUSSIAN 09 program.^54^ Their nature of convergence was verified *via* frequency calculation, which in return affirmed all of the frequencies to be real. Both the frontier-molecular orbitals and the molecular-electrostatic potential surfaces were generated from the formatted check (Fchk) file of the optimized structures. All of the measurements were accomplished using the density functional theory (DFT) approach with the B3LYP method,^55,56^ while using 6-31g(d,p) as the basis set.

## 3. Results and discussion

### 3.1. Mesomorphic behavior of the investigated Sn derivatives

**Fig. 2:**
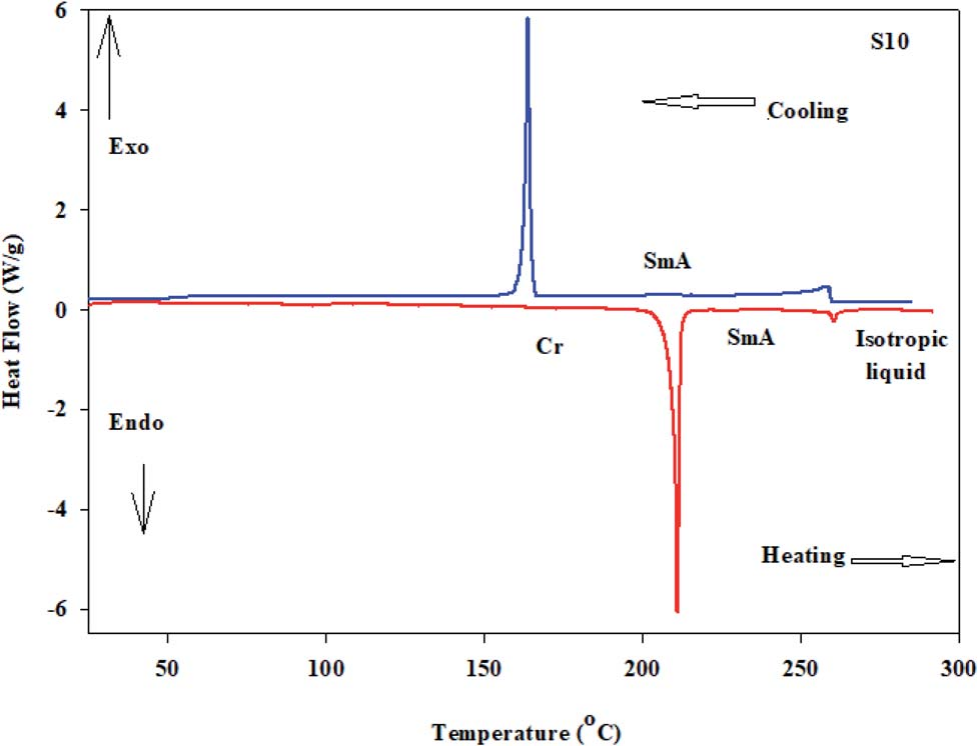
DSC thermograms of compound S10 at a rate of ±10 °C min^−1^ recorded from heating and cooling scans.

**Fig. 3:**
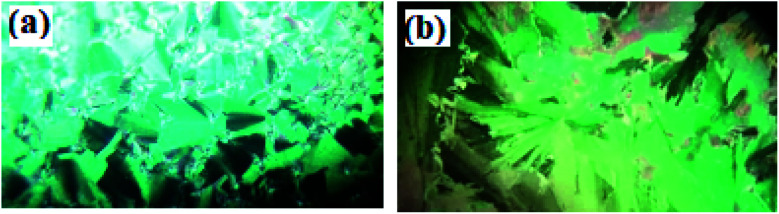
The focal conic textures of SmA phases observed under POM for the materials (a) S6 at 240.0 °C and (b) S12 at 260.0 °C.

**Fig. 4:**
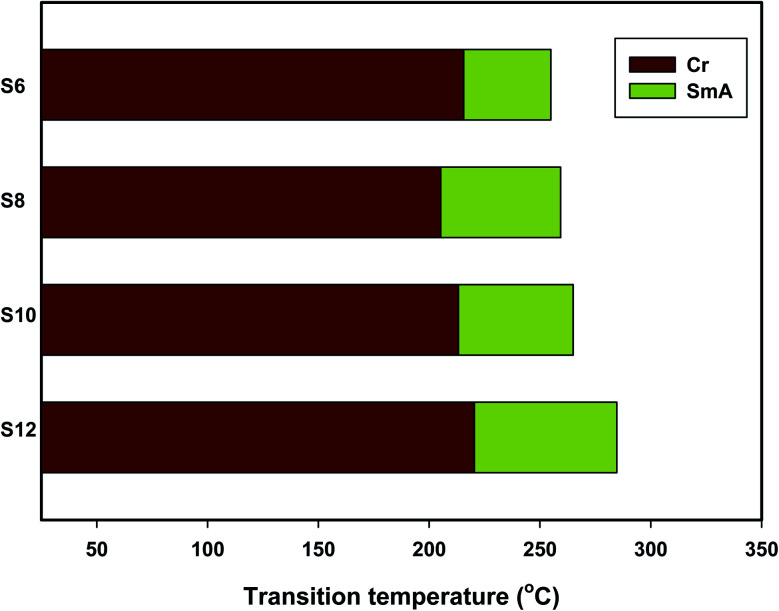
Effects of the terminal alkoxy chain length (*n*) on the mesomorphic transitions of the presented Sn homologues.

Mesomorphic characteristics of the synthesized benzene sulphonic acid derivatives (Sn) have been investigated by DSC and POM. The DSC thermograms of compound S10 during both heating/cooling scans as a representative example are displayed in Fig. 2. It is observed that upon heating, the materials showed two endothermic peaks of the crystal–smectic A and smectic A–isotropic transitions and these were reversed upon cooling. Optical images of the S6 and S12 derivatives under POM are illustrated in Fig. 3. The SmA mesophase showed a focal conic fan texture, which was identified upon heating and cooling scans. The POM measurements confirmed the DSC analyses. This indicates that these derivatives exhibited monomorphic enantiotropic properties. In general, the transition characteristic observation changes according to the molecular geometry of the prepared materials. Significant endothermic as well as exothermic transitions were observed dependent on the attached terminal flexible chain length group and this could be ascribed to the mesomorphic transitions. Details of the transition temperature results and the enthalpy of transitions for the entire investigated Sn series, as derived from DSC measurements, are listed in Table 1. The transition temperatures of all of the evaluated compounds are graphically depicted in Fig. 4 in order to illustrate the impact of terminal alkoxy-chain length (*n*) on their mesomorphic behavior.

**Table 1:** Mesophase transition temperature (°C), enthalpy of transition Δ*H* (kJ mol^−1^), mesomorphic range Δ*T* (°C), and normalized entropy of transition Δ*S*/*R* values for the presented Sn homologues^a^

Comp.	*T*_Cr-SmA_	Δ*H*_Cr-SmA_	*T*_SmA-I_	Δ*H*_SmA-I_	Δ*T*	Δ*S*_SmA-I_/*R*
S6	215.6	37.89	254.9	2.80	39.3	0.64
S8	205.2	34.08	259.3	2.21	54.1	0.50
S10	213.2	58.01	265.0	2.03	51.8	0.45
S12	220.4	47.62	284.7	3.10	64.3	0.67

a Cr-SmA = solid to smectic A mesophase transition. SmA-I = smectic A to isotropic liquid mesophase transition.

Table 2 and Fig. 4 show that the melting temperatures (Cr-SmA) of the prepared Sn series homologues exhibit an irregular trend, which is less sensitive to the length of the terminal alkoxy chain (*n*). The melting transition increases as the polarizability of the molecule within the same series increases. However, the observed current trend was not in agreement with this common rule. All synthesized members of the group are mesomorphic in nature with high mesomorphic thermal transitions and a monomorphic enantiotropic mesophase range depending on their flexible terminal length. All of the compounds are purely smectogenic and possess SmA phase with a high thermal stability that increases with the terminal alkoxy chain length.^57,58^ Compound S6 exhibits the SmA phase with the lowest thermal stability and a temperature range of 254.9–39.3 °C. In the case of the S8 derivative, it possesses an enantiotropic SmA phase with stability and a temperature range close to 259.3–54.1 °C. Also, the S10 homologue exhibits smectogenic stability and a temperature range of nearly 265.0–51.8 °C. However, the derivative bearing the longest terminal chain length (S12) has the highest SmA stability and a broadened temperature range (284.7–64.3 °C). Generally, the molecular geometry, polarizability and dipole moment of the examined materials are highly impacted by the mesomeric-nature of the attached wing groups. Moreover, the mesomorphic character is enhanced by the elevation of the mesogenic core polarity. The mesophase range of the present investigated series increased in the order: S12 > S8 > S10> S6. The mesophase phenomena of calamitic molecules are a direct impact of molecular–molecular interactions that essentially depend on the geometrical shape of the terminal polar groups. The increment of the van der Waals attraction forces between the long terminal alkoxy chains facilitates their lamellar packing and formation of the smectic A phase.

**Table 2:** Thermal energy, enthalpy, Gibb's free energy, entropy, ionization potential, and electron affinity data for the series at room temperature calculated at the B3LYP/6-31g(d,p) level

Compound	ZPE, kcal mol^−1^	Thermal, kcal mol^−1^	Enthalpy, kcal mol^−1^	Gibb's free energy, kcal mol^−1^	Entropy, kcal mol K^−1^	I.E., eV	E.A., eV
S6	305.498	326.236	326.829	259.805	224.800	5.919	2.526
S8	341.375	363.801	364.393	293.404	238.098	6.300	2.050
S10	377.129	401.288	401.881	326.490	252.860	6.294	2.050
S12	412.887	438.757	439.349	359.548	267.656	6.295	2.050

The normalized entropy changes (Δ*S*/*R*) of mesophase transitions were calculated for the investigated homologues (Sn) and highlighted in Table 1. Irregular trends with small values of Δ*S*/*R* associated with the SmA-isotropic transition were observed. This could be attributed to the molecular biaxiality induced by the –COO- linking moiety and the relatively high magnitude of the clearing-temperatures, which in return decreased the SmA-isotropic entropy changes.^59–61^

### 3.2. Computational studies

#### 3.2.1. Thermal and geometrical parameters

**Fig. 5:**
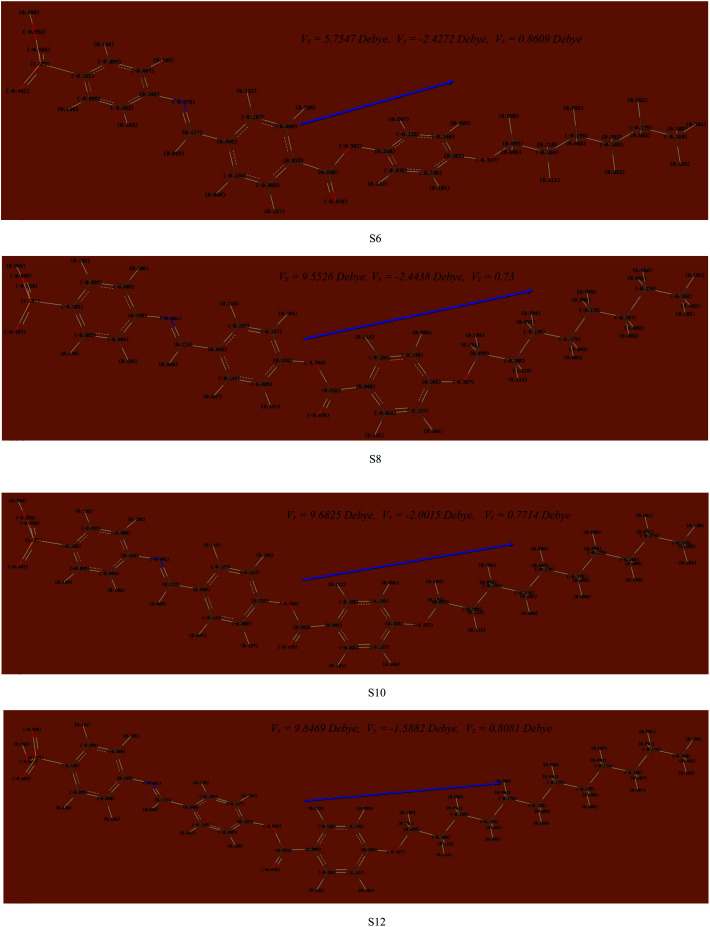
Atomic charges and dipole moment vectors for the optimized structures of the presented Sn series, predicted at the B3LYP/6-31g(d,p) level.

Computational calculations using DFT were carried out for all of the synthesized compounds (Sn) in order to correlate the estimated quantum chemical parameters and the experimental findings. The theoretical DFT calculations were performed in the gas phase with the DFT/B3LYP method at basis set 6-31G(d,p). Since the synthesized homologues (Sn) are mesomorphic, they should present in a planar conformation. The optimized structure of each member of the present series was affirmed to be stable by the frequency calculation for which no imaginary frequency was predicted for any member (Fig. 5). The zero-point energy and other calculated quantum thermal parameters are highlighted in Tables 2 and 3. They were predicted to increase with the increasing size of the molecule. In the same vein, the Mulliken atomic charges and dipole moment vectors, which are reactivity predictors, are illustrated in Fig. 5. The vector arrow points in the direction of the alkoxy chain for all of the derivatives, thus suggesting the electron donating tendency of the alkoxy terminal owing to its *para* position. Moreover, the polarity of the derivatives was predicted to increase with the size of the system as indicated by the increasing magnitude of the dipole moment vectors with the alkoxy chain length. On the other hand, it could be seen from Tables 2, 3 and Fig. 5 that all of the derivatives are not completely linear with a slight twisting angle in the core structure. Moreover, the length of the terminal flexible chains has no significant effect on the aromatic ring planarity. It was found that^62,63^ the planarity of the mesogenic moiety of the mesomorphic compounds is influenced by the electronic nature of the polar attached substituent. Hence, the conjugated π-cloud interactions obtained from the sulphanilic acid moiety play an important role that offers a high thermal stability with good geometrical parameters in the present investigated system.

**Table 3:** HOMO energy, LUMO energy, orbital energy gap, dipole moment, and polarizability data calculated at the B3LYP/6-31g(d,p) level

Compound	*E*_HOMO_, eV	*E*_LUMO_, eV	Δ*E*, eV	Dipole moment, debye	Polarizability, bohr^3^
S6	−5.919	−2.526	3.393	6.305	370.67
S8	−6.300	−2.050	4.250	9.888	398.77
S10	−6.294	−2.050	4.244	9.917	421.86
S12	−6.295	−2.050	4.245	10.007	444.79

The estimated theoretical data were correlated with the experimental variables of the mesomorphic stability as well as the length of the terminal alkoxy chains (*n*). It is well known that the mesomorphic stability and kind of mesophase of liquid crystalline materials is mainly dependent on the length of the terminal flexible groups, and this is most often accounted for in terms of molecular geometry.^64^ Moreover, the polarity of the incorporated substituents, polarizability, rigidity, and shape of the LC molecule are considered important parameters for the enhancement of the type and thermal stability of the mesophases formed. On the other hand, the lower ionization potential (I.E., Table 2) calculated for the shortest terminal length derivative (S6) indicates a more basic nature than others in the series.^65^ Moreover, a pronounced increment of the dipole moment and polarizability listed in Table 3 are observed with increasing smectic A stability as the length of the alkoxy chain (*n*) of our investigated compounds (Sn) increases from *n* = 6 to *n* = 12 carbons. The rise in dipole moment with increasing number of CH_2_ in the terminal alkoxy group is in good agreement with the previous report by Davies for the dipole moments of alkyl chlorides in the vapor state.^66^ The increment of polarizability with terminal length (*n*) may be attributed to the aspect ratio of the molecules. As the molecular structure aspect ratio increases, the space-filling of the liquid crystalline compound increases, and this results in the enhancement of the polarizability. Furthermore, the dilution of the core–core interactions as the alkoxy chain length increases will influence the polarizability of the whole molecule and increases the intermolecular adhesion forces between molecules, which promotes the degree of molecular ordering and formation of smectic mesophases.

#### 3.2.2. Frontier molecular orbitals (FMOs)

**Fig. 6:**
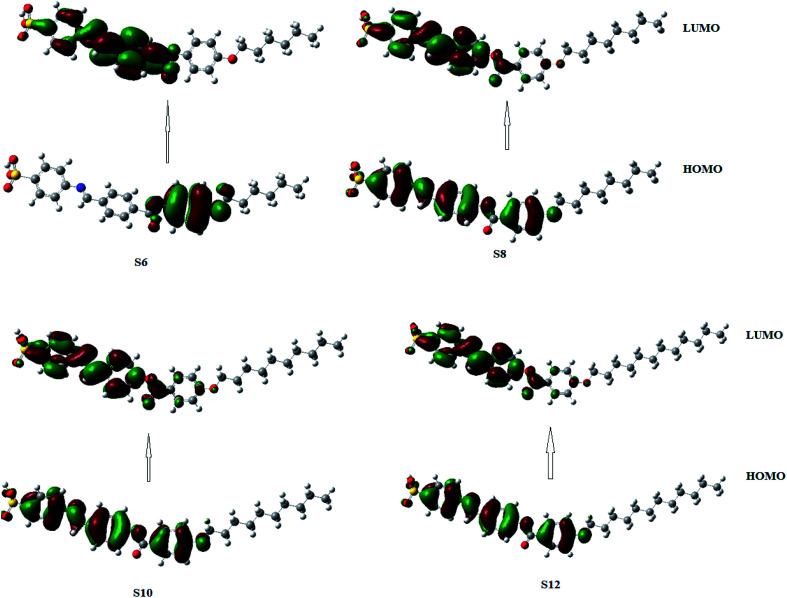
Estimated FMOs for the synthesized Sn series of homologues.

For the present investigated compounds (Sn), the frontier molecular orbitals HOMO (highest occupied) and LUMO (lowest unoccupied) analysis depicting the distribution of the HOMO and LUMO in the compounds is presented in Fig. 6 and their resulting energies, as well as energy gaps, are listed in Table 3. The energy gap (Δ*E*) between the HOMO and LUMO levels is an indicator of the chemical reactivity of the compounds.^67^ The lower its value, the more reactive the molecule would be. The predicted energy gap included in Table 3 affirms the S6 derivative to be more reactive than the others. It is also softer than others as the energy gap is inversely related to the softness.^68^ Moreover, in the HOMO of S6, the electron clouds are evenly distributed over the carbon atoms and the π-electron of the terminal phenyl ring attached to the alkoxy chain, but for the others in the series, the distribution extended to the other two phenyl rings. In addition, the similar electron cloud distribution predicted for the HOMO of S8, S10 and S12 could be attributed to their approximately equal HOMO energies. For the LUMO, all of the compounds (Sn) showed similar electron cloud distributions over the carbon atoms and the π-system of the first two phenyl rings attached to the Schiff base linker with the third phenyl ring attached to the alkoxy chain group of S6 being more electron deficient than that of the other counterparts.

#### 3.2.3. Molecular electrostatic potential (MEP)

**Fig. 7:**
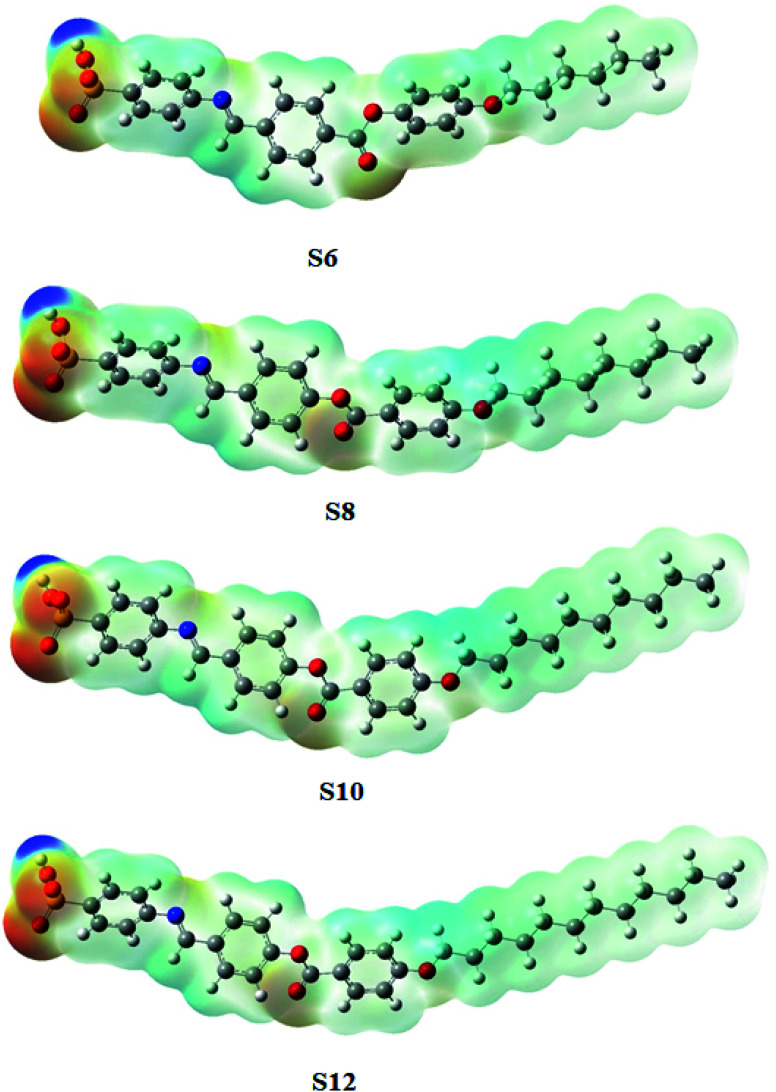
Molecular electrostatic potential (MEP) plots of the Sn series.

The molecular geometry of the prepared liquid crystalline material is impacted by the mesomeric-configurations, which are normally affected by the molecular–molecular interactions. One of the best methods for the estimation of the presence of inter or intra-molecular interactions of the evaluated molecules is the MEP. So, the MEP is an indicator that shows the distribution of electron density within a molecule. The MEP for the synthesized homologous series, Sn, was theoretically predicted under the same B3LYP/6-31g(d,p) level of calculation according to MEP (Fig. 7). The MEP analysis predicts low electron density and high electrostatic-potential for the sulphanilic hydrogen atom of all of the investigated compounds in the series (Sn) as it is being shadowed by a blue cloud. On the other hand, the red cloud encircling the oxygen atoms of the sulphanilic acid moiety indicates high electron density. In addition, an appreciable electron density could be noticed for the carbonyl oxygen of the homologous series.

### 3.3. Electric properties

**Fig. 8:**
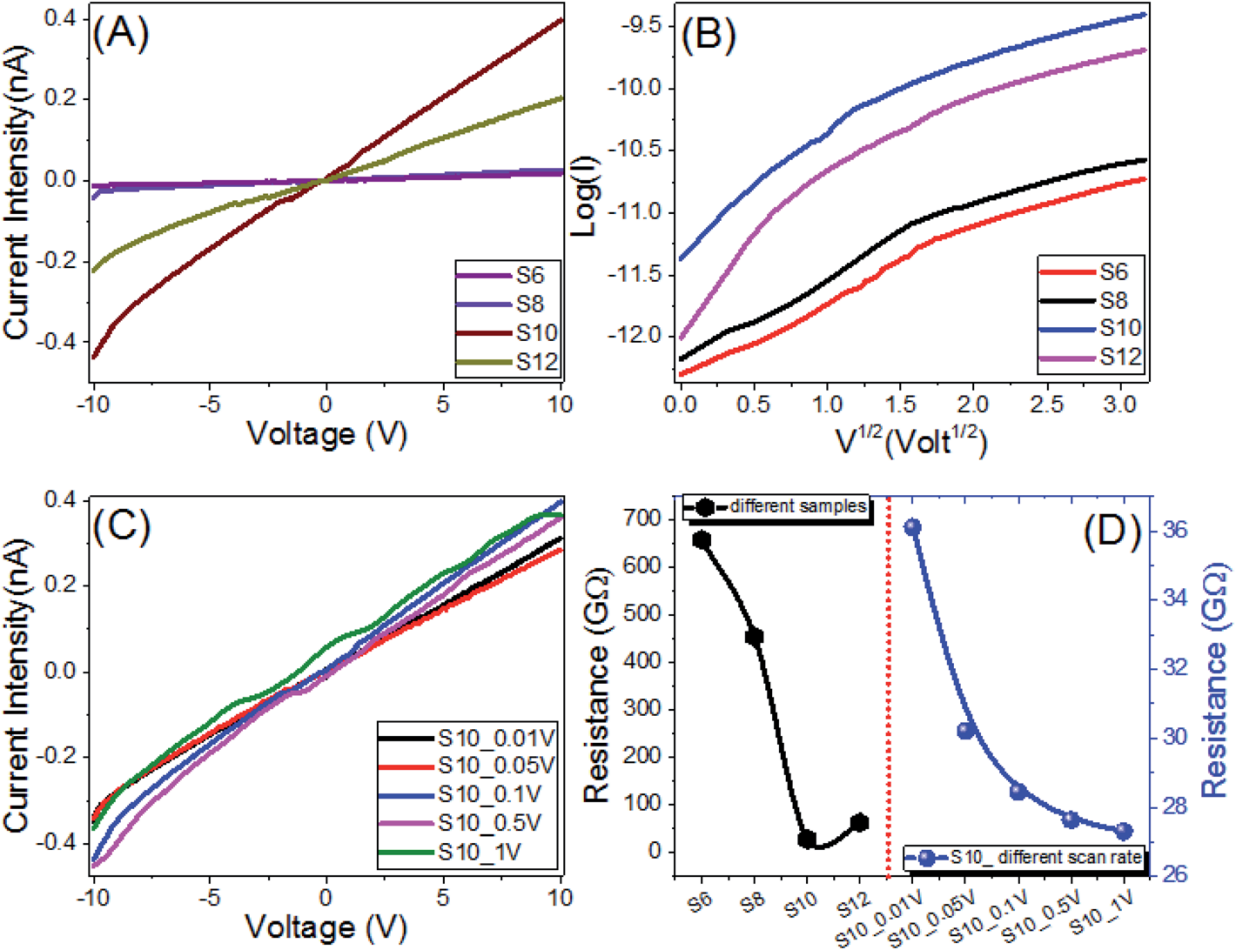
Electrical characteristics of the Sn series: (A) current–voltage characteristics of the Sn series, (B) log(*I*) *vs. V*^0.5^ plots for the S10 sample at different step scans, (C) current–voltage characteristics of the S10 sample at different step scans, and (D) electrical resistance for the Sn samples and an individual S10 sample at different step scans.

The electrical properties and current–voltage (*I*–*V*) characteristics of the investigated Sn series, are measured from −10 V to 10 V with different scan steps, 1 V to 0.005 V, and presented in Fig. 8A and C. The dimensions of the electrode were 22 mm × 22 mm × 0.03 mm. The behaviors are almost linear (ohmic behaviors). As a result, the materials' resistances are nearly constant and independent of the current flowing through them. Recent research has discovered that at low voltage, polymeric and organic systems behave like Schottky diodes. But in the present investigation, the relation between log(*I*) and *V*^1/2^ is non-linear as illustrated in Fig. 8B, which implies that our Sn electrodes do not follow the Schottky diode behavior. Fig. 8A shows how increasing the applied voltage and increasing the carbons to *n* = 10 increased the current intensity to 0.4 nA at 10 V for the S10 film. As the scan step increased to 1 V, the current intensity is slightly increased, Fig. 8C. As shown in Fig. 8C, the current depends on the voltage and the scan rate. So, the sample seems to have a capacity of some kind. It could be electronic or perhaps ionic. At a specific voltage, the resistive current only depends on voltage and not on the scan rate, while the capacitive current mainly depends on the scan rate. At a given voltage, the capacitance can be calculated by dividing the difference in current by the difference in scan rate, *i.e.*, *C* = (*I*_1_ − *I*_2_)/([d*V*_1_/d*t*] − [d*V*_2_/d*t*]) at a specific voltage. The obtained mean value of the capacitance is 148.7 μF for S10 at 5 V. The resistance of the Sn series is decreased by increasing the number of carbons to *n* = 10. The value of the resistance is decreased from 658.5 GΩ for S6 to 284.4 GΩ for S10. The electrical resistance of the S10 film is decreased from 361.0 GΩ to 273.0 GΩ by increasing the scan step from 0.01 V to 1 V as shown in Fig. 8D. The values of the electrical conductance (*σ*) were obtained and are shown in Fig. S2† (ESI†). The value of the electrical conductance is increased from 1.52 pS to 35.16 nS by increasing the number of carbons from 6 to 10, Table 4, since the electrical conductance depends mainly on the number and mobility of charge carriers.^69–71^ By increasing the scan step from 0.01 to 1 V, the film conductance is increased from 27.7 to 36.6 pS. The high resistances and energy band gaps (Section 3.4) imply that the compounds under investigation are dielectrics, with ionic impurities likely causing the materials' conductivity. The compositional heterogeneity of different mobility of these impurities is considered as a potential enhancement of the electrical properties.^72,73^

**Table 4:** Values of the electrical conductance (*σ*), energy gap (*E*_g_), and Urbach energy (*E*_U_) for the Sn series

Sample	*σ* (pS)	*E*_g_ (eV)	*E*_U_ (meV)	S.D.	*R* ^2^
S12	15.97	3.75	592.0	9.53	0.9979
S10	35.16	3.58	1261.1	34.59	0.9947
		3.23	502.4	17.29	0.9906
S8	2.20	3.40	1489.3	39.14	0.9958
S6	1.52	3.30	2136.6	67.66	0.9930

### 3.4. Optical spectra and energy gap calculations

**Fig. 9:**
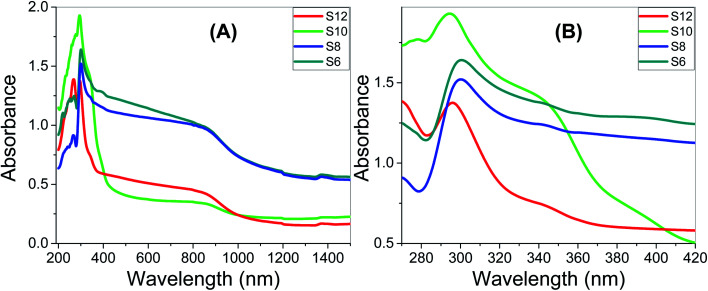
Optical absorbance spectra of **Sn** films; (A) wavelength range 200–1500 nm; (B) wavelength range 270–420 nm.

Fig. 9A and B show how the absorbance spectra of the Sn films are affected by the wavelength of the incident light and the number of carbons of the Sn series. Analysis of the absorbance spectra of S6, S8, and S12, depicted in Fig. 9, show that Sn has a high absorption behavior. For the present Sn series, all of the films display strong absorbance in the UV region and the strongest absorbance was observed for S10 up to 350 nm, Fig. 9A. The absorbance then drops to a plateau up to 880 nm, before dropping again to a minimum absorbance around 1282 nm. Fig. 9C shows a strong absorption band at 298 and 294 nm for S6 and S12, which is blue-shifted by increasing the number of carbons of the prepared Sn series. The S10 sample showed two convoluted absorption bands centered at 293 nm and 344 nm. The absorbance intensity is in the order S10 > S8 > S12. The right edge of the absorption band is blue-shifted by increasing the number of carbons in the Sn series. This red-shift is mainly attributed to the size effects, where large size increases the spin–orbit coupling and controls the exciton positions.^74^ This strong absorption and wide absorption band in the UV/vis region is a desirable feature for designing energy-efficient solar cells.^75^ The transmittance spectra of the Sn films, Fig. S3 (ESI†), are affected by the wavelength of the incident light and the number of carbons of the Sn series. All films showed transmission less than 10% up to 360 nm, and then the transmission increased to reach a plateau in the visible light region. Then, the transmission increased exponentially in the near IR region to reach maxima of ∼12.4%, 13.2%, 46.7%, and 57.1% at 1315 nm for the Sn films. After that, the transmission slightly decreased as the wavelength increased.

According to the optical absorption theorem, the relationship between absorption coefficient, *α*_a_, and the photon energy, *E*_ph_ = *hν*, *h* = 6.625 × 10^−34^ J s^−1^, for the direct allowed transition is given by:^76^

**Fig. 10:**
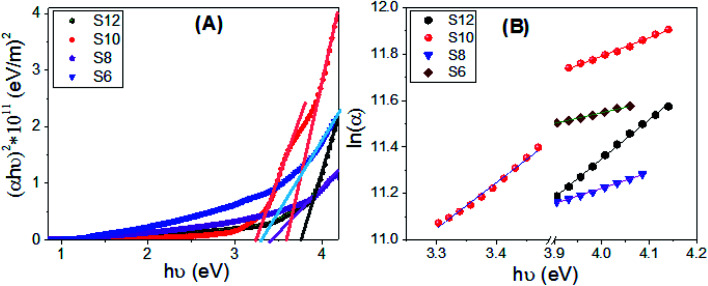
Calculations of (A) the energy gap and (B) Urbach energy values for S6, S8, S10, and S12 films.

1 (*α*_a_*E*_ph_)^2^ = *A*(*E*_ph_ − *E*_g_)

where *E*_g_ is the optical energy gap. The values of direct *E*_g_ for S6, S8, S10, and S12 are obtained by extending the linear segments of the plot of (*α*_a_*E*_ph_)^2^*vs. E*_ph_ to zero as shown in Fig. 10A. The linear part observed for this figure indicates that the transition is performed directly. Interestingly as reported in Table 4, there are two direct band gaps for S10, but one for S6, S8, and S12. The material S10 may be proceeded to afford a mixture of two geometrical isomers (*E* and *Z*) and thus, two band gaps of energy were observed during measurements.^77,78^ Meanwhile, the other compounds in the series have observed only one isomer with a percent ratio of 100 : 0 for the *E* and *Z* isomers. The values of the band gaps for S10 are 3.23 and 3.58 eV, which are suitable for solar energy applications.^11–15^ The energy gap is increased from 3.3 eV to 3.75 eV by increasing the terminal chain length from S6 to S12. This increase is ascribed to the influence of the density of localized states. This behavior is consistent with the previously reported studies.^79^ The strong absorption and the extension of the bandgap edges are very important for solar energy applications, especially photoelectrochemical hydrogen generation, and solar cells.^80–82^

Urbach energy (*E*_U_) refers to the disorder in the material and represents the width of the exponential absorption edge (Urbach tail of the valence and conduction bands).^81^ The exponential dependency of *E*_U_ can be determined according to the following equation:^83^

2 *α*_a_ = *α*_ao_ exp(*E*_ph_/*E*_u_) → *E*_u_ = δ*E*_ph_/δ(ln(*α*_a_))

where *α*_ao_ is the band tail parameter that can be given by:^84^

3 *α*_ao_ = (4π*σ*_o_/*x*Δ*E*_c_)^1/2^

where *c* is the speed of light, *σ*_o_ is the electrical conductivity at absolute zero, and Δ*E* represents the width of the tail of the localized state in the forbidden gap. Fig. 10B shows the plot of ln(*α*) *vs. hν* for the two band gaps of S6, S8, S10, and S12. The values of *E*_U_ were obtained from the slopes of the linear fitting of these curves and reported in Table 4. The statistical parameters, standard deviation (S.D.) and correlation coefficient (*R*^2^) are also reported in this table. The values are 592.0 ± 9.53 for S12 and 1261.1 ± 34.59 and 502.4 ± 39.14 eV for S10, which refers to the extension of the bandgap edges to cover a wide range of the spectral range. For future work and based on the obtained optical properties (absorption, *E*_g_ and *E*_U_), the designed liquid crystalline materials can be further enhanced by incorporating a conductive plasmonic nanomaterial to improve the conductivity and reduce the bandgap.

## 4. Conclusions

A novel mesomorphic series based on a terminal sulphonic acid moiety, namely (*E*)-4-((4-((4-(alkoxy)benzoyl)oxy)benzylidene)amino)benzenesulfonic acid (Sn), was synthesized and evaluated for its potential in solar energy applications. Molecular structure elucidation was carried out *via* elemental analysis, FT-IR spectroscopy, and NMR spectroscopy. Mesomorphic investigations of the prepared homologues were conducted using DSC and POM. An examination of the DSC and POM data revealed that all of the presented synthesized derivatives are monomorphic, possessing a smectogenic (SmA) mesophase with enantiotropic behavior. In addition, all set members have a high thermal transition temperature. The high polarity of the terminal –SO_3_H group aided in the stabilization of the molecules, which made them achieve high thermal SmA stability. Computational DFT calculations corroborated the recorded experimental values of the mesophase behavior. Moreover, the terminal sulphonic moiety has an essential impact on the thermal and physical parameters of the possible geometries. Energy conversion device measurements affirmed that all of the investigated derivatives exhibit ohmic behavior with electric resistance in the GΩ range, as indicated by electrical property measurements. The highest electrical conductivity, 35.16 pS, was reported for the derivative S10. The energy gap increased from 3.3 eV to 3.75 eV upon increasing the terminal chain length from *n* = 6 (S6) to *n* = 12 (S12). Two band gaps were reported for compound S10, 3.23 and 3.58 eV, with band edge tails of 1261.1 ± 34.59 and 502.4 ± 39.14 eV, respectively.

## Conflicts of interest

The authors declare no conflicts of interest.

## Supplementary Material

RA-011-D1RA02517A-s001
