# Supplementary material for: Novel sulphonic acid liquid crystal derivatives: experimental, computational and optoelectrical characterizations
Source: RSC Adv. 2021 Aug 17;11(45):27937–49. doi: 10.1039/d1ra02517a (PMC9039420; doi:10.1039/d1ra02517a)
Supplement: RA-011-D1RA02517A-s001 [file RA-011-D1RA02517A-s001.pdf]

Electronic Supplementary Material (ESI) for RSC Advances.
This journal is © The Royal Society of Chemistry 2021

Supplementary Materials

Novel Sulphonic Acid Liquid Crystal Derivatives; Experimental, Computational and Optoelectric Characterizations

Latifah A. Alshabanah[1], Laila A. Al-Mutabagani[1], Sobhi M. Gomha[2,3*], Hoda A. Ahmed[3,4*], Saheed A. Popoola[2] and Mohamed Shaban[5,6]

[1]Department of Chemistry, College of Science, Princess Nourah bint Abdulrahman University, Riyadh, Saudi Arabia.

[2]Chemistry Department, Faculty of Science, Islamic University in Almadinah Almonawara, Almadinah Almonawara, 42351, Saudi Arabia

[3]Department of Chemistry, Faculty of Science, Cairo University, Cairo 12613, Egypt, ahoda@sci.cu.edu.eg, sm.gomha@iu.edu.sa

[4]Chemistry Department, College of Sciences, Yanbu, Taibah University, Yanbu 30799, Saudi Arabia.

[5]Department of Physics, Faculty of Science, Islamic University in Almadinah Almonawara, Almadinah, 42351, Saudi Arabia

[6] Nanophotonics and Applications Labs, Department of Physics, Faculty of Science, Beni-Suef University, Beni-Suef 62514, Egypt

** Correspondence: (SM.G) sm.gomha@iu.edu.sa* and (HA.A) *ahoda@sci.cu.edu.eg*

---

1. *Materials*

4-Hexyloxybenzoic acid, 4-octoyloxybenzoic acid, 4-decyloxybenzoic acid, 4-dodecyloxybenzoic acid 4-hydroxybenzaldehyde, and 4-aminobenzenesulfonic acid, were purchased from Sigma Aldrich (Germany) while the dichloromethane, *N,N′*-dicyclohexylcarbodiimide (DCC), ethanol and 4-dimethylaminopyridine (DMAP) were purchased from Aldrich (Wisconsin, USA).

2.2.2. Synthesis of (E)-4-((4-((4-(alkoxy)benzoyl)oxy)benzylidene)amino) benzenesulfonic acid, Sn

A mixture of imine compound **3** (2.77g, 10 mmol) and the appropriate 4-alkoxy benzoic acid derivatives **4** (10 mmol for each) in dry methylene chloride (25 mL) containing *N*, *N′*–dicyclohexylcarbodiimide (DCC, 10 mmol) and few crystals of

4–dimethylaminopyridine (DMAP), as catalyst, were left to stand for 72 hours at room temperature with continuous stirring. The solid separated was then filtered off and the solution evaporated. The solid residue obtained was recrystallized from ethanol to give TLC pure products. The purity of the prepared samples was checked with thin-layer chromatography (TLC) using TLC sheets coated with silica gel (E Merck), and CH_2Cl_2/CH_3OH (9:1) as eluent, whereby only one spot was detected by a UV-lamp. Infrared spectra (IR), ^{1}H-NMR, and elemental analyses for compounds investigated were consistent with the structures assigned. ^{1}H-NMR data showed the expected integrated aliphatic to aromatic proton ratios in all compounds investigated.

The physical data of products **Sn** are listed below:

(E)-4-((4-((4-(Hexyloxy)benzoyl)oxy)benzylidene)amino)benzenesulfonic acid (S*6).*
Yield: 92.3%; mp 216.0 °C, FTIR (ύ, cm^{-1}): 3423 (OH), 3034, 2944, 2928, 2917 (C-H), 1733 (C=O), 1626 (C=N), 1574 (C=C), 1346 (S=O stretching). ^{1}H-NMR (400 MHz, DMSO): δ/ppm: 0.84 (s, 1H, SO_3H), 0.96-1.02 (t, 3H, $\underline{CH_3}(CH_2)_3CH_2CH_2O$-), 1.06-1.57 (m, 6H, $CH_3\underline{(CH_2)_3}CH_2CH_2O$-), 1.66-1.78 (m, 2H, $CH_3(CH_2)_3\underline{CH_2}CH_2O$-), 4.00-4.02 (t, 2H, $CH_3(CH_2)_3CH_2\underline{CH_2}O$-), 6.71-6.73 (d, 2H, Ar–H), 6.98-7.00 (d, 2H, Ar–H), 7.49-7.51 (d, 2H, Ar–H), 7.64-7.66 (d, 2H, Ar–H), 7.89-7.91(d, 2H, Ar–H), 8.09-8.12 (d, 2H, Ar–H), 10.08 (s, 1H, CH=N) ppm; ^{13}C-NMR (400 MHz, DMSO): δ/ppm: 13.94, 22.09 (CH_3), 25.16, 25.32, 28.56, 32.29 (CH_2), 67.74 (CH_2-O), 106.83, 114.01, 119.18, 125.94, 126.8, 128.50, 129.63, 135.32, 143.14, 144.52 (Ar-C), 155.37, 156.62 (C=N), 161.13 (Ar-C-OR), 164.81 (C=O) ppm. Anal. Calcd. for $C_{26}H_{27}NO_6S$ (481.56): C, 64.85; H, 5.65; N, 2.91. Found: C, 64.70; H, 5.52; N, 2.81%.

(E)-4-((4-((4-(Octyloxy)benzoyl)oxy)benzylidene)amino)benzenesulfonic acid (S8).
Yield: 89.1%; mp 205.0 °C, FTIR (ύ, cm^{-1}): 3414 (OH), 3031, 2946, 2922, 2918 (C-H), 1730 (C=O), 1612 (C=N), 1551 (C=C), 1352 (S=O stretching). ^{1}H-NMR (400 MHz, DMSO): δ/ppm: 0.82 (s, 1H, SO_3H), 0.97-1.03 (t, 3H, $\underline{CH_3}(CH_2)_5CH_2CH_2O$-), 1.02-1.55 (m, 10H, $CH_3\underline{(CH_2)_5}CH_2CH_2O$-), 1.69-1.73 (m, 2H, $CH_3(CH_2)_5\underline{CH_2}CH_2O$-), 3.99-4.01 (t, 2H, $CH_3(CH_2)_5CH_2\underline{CH_2}O$-), 6.70-6.72 (d, 2H, Ar–H), 6.96-6.99 (d, 2H, Ar–H), 7.35-7.38 (d, 2H, Ar–H), 7.60-7.62 (d, 2H, Ar–H), 7.77-7.79 (d, 2H, Ar–H), 8.02-8.05 (d,

2H, Ar–H), 9.85 (s, 1H, CH=N) ppm. Anal. Calcd. for $C_{28}H_{31}NO_6S$ (509.61): C, 65.99; H, 6.13; N, 2.75. Found: C, 65.74; H, 6.02; N, 2.59%.

(E)-4-((4-((4-(Decyloxy)benzoyl)oxy)benzylidene)amino)benzenesulfonic acid (S10).

Yield: 88.2%; mp 213.0 °C, FTIR (ύ, cm^{-1}): 3418 (OH), 3027, 2944, 2928, 2917 (C-H), 1731 (C=O), 1626 (C=N), 1575 (C=C), 1346 (S=O stretching). ^{1}H-NMR (400 MHz, DMSO): δ/ppm: 0.81 (s, 1H, SO_3H), 0.96-1.02 (t, 3H, $\underline{CH_3}(CH_2)_7CH_2CH_2O$-), 1.06-1.57 (m, 14H, $CH_3\underline{(CH_2)_7}CH_2CH_2O$-), 1.66-1.78 (m, 2H, $CH_3(CH_2)_7\underline{CH_2}CH_2O$-), 4.01-4.03 (t, 2H, $CH_3(CH_2)_7CH_2\underline{CH_2}O$-), 6.57-6.59 (d, 2H, Ar–H), 6.87-6.89 (d, 2H, Ar–H), 6.98-7.00 (d, 2H, Ar–H), 7.49-7.51 (d, 2H, Ar–H), 7.89-7.91(d, 2H, Ar–H), 8.06-8.07 (d, 2H, Ar–H), 9.74 (s, 1H, CH=N) ppm; ^{13}C-NMR (400 MHz, DMSO): δ/ppm: 15.53 (CH_3), 22.85, 24.95, 25.84, 27.17, 29.01, 30.92, 32.14, 33.83 (CH_2), 66.91 (CH_2-O), 107.25, 116.38, 125.12, 127.31, 129.51, 131.82, 132.64, 136.89, 145.07, 148.68 (Ar-C), 156.00, 158.31 (C=N), 159.74 (Ar-C-OR), 164.86 (C=O) ppm. Anal. Calcd. for $C_{30}H_{35}NO_6S$ (537.67): C, 67.02; H, 6.56; N, 2.61. Found: C, 67.17; H, 6.50; N, 2.43%.

(E)-4-((4-((4-(Dodecyloxy)benzoyl)oxy)benzylidene)amino)benzenesulfonic acid (S12).

Yield: 90.6%; mp 221.0 °C, FTIR (ύ, cm^{-1}): 3424 (OH), 3029, 2941, 2928, 2916 (C-H), 1724 (C=O), 1626 (C=N), 1554 (C=C), 1347 (S=O stretching). ^{1}H-NMR (400 MHz, DMSO): δ/ppm: 0.80 (s, 1H, SO_3H), 0.97-1.02 (t, 3H, $\underline{CH_3}(CH_2)_9CH_2CH_2O$-), 1.06-1.55 (m, 18H, $CH_3\underline{(CH_2)_9}CH_2CH_2O$-), 1.61-1.76 (m, 2H, $CH_3(CH_2)_9\underline{CH_2}CH_2O$-), 3.92-3.98 (t, 2H, $CH_3(CH_2)_9CH_2\underline{CH_2}O$-), 6.50-6.52 (d, 2H, Ar–H), 6.80-6.82 (d, 2H, Ar–H), 6.96-6.98 (d, 2H, Ar–H), 7.42-7.45 (d, 2H, Ar–H), 7.80-7.82 (d, 2H, Ar–H), 8.00-8.03 (d, 2H, Ar–H), 9.69 (s, 1H, CH=N) ppm. Anal. Calcd. for $C_{32}H_{39}NO_6S$ (565.72): C, 67.94; H, 6.95; N, 2.48. Found: C, 67.79; H, 6.74; N, 2.25%.

2.2. Characterization

Perkin-Elmer B25 (Perkin-Elmer, Inc., Shelton, CT USA) spectrophotometer was used for infrared spectra measurements. Varian EM 350L 500 MHz spectrometer (Oxford, UK) was used for recording ^{1}HNMR spectra using tetramethyl silane as internal standard in $CDCl_3$; the chemical shift values recorded as δ (in ppm units).

Thermo Scientific Flash 2000 CHS/O Elemental Analyzer, Milan, Italy was used for Elemental analyses.

TA Instruments Co. (Q20 Differential Scanning Calorimeter, DSC; USA) was used for recording phase transitions. DSC calibration was carried out using lead and indium to calibrate the melting temperatures and enthalpies. Samples of 2–3 mg were used in aluminum pans for DSC investigation. The heating rate was 10°C/min in nitrogen gas as an inert atmosphere (30 ml/min). All transitions temperatures were measured from the second heating scan.

Transition temperatures for the prepared compounds were checked and phases identified by Polarized optical microscope (POM, Wild, Germany) attached with Mettler FP82HT hot stage.

The optical absorbance and transmission spectra of the investigated array, **Sn**, were measured using a Perkin Elmer spectrophotometer (Lambda 950 UV-VIS-NIR) over a wavelength range of 250 to 1500 nm. A Keithley measurement source device (Model 4200 SMU) was used to test the electrical properties of the examined films. The current-voltage (I–V) characteristics of the Sn series were recorded by varying the applied voltage (V) from -10 V to 10 V with different scan steps, 1V to 0.005V.

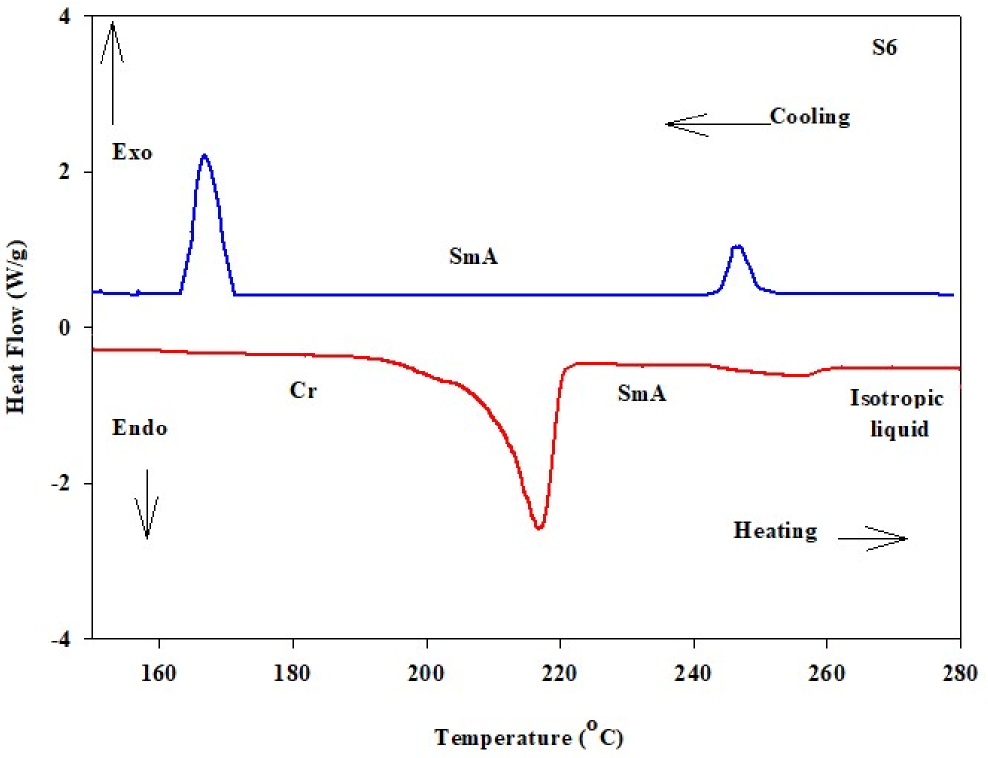

Figure S1. DSC thermograms of compounds **S6** at a rate of ±10°C/min recorded from heating and cooling scans.

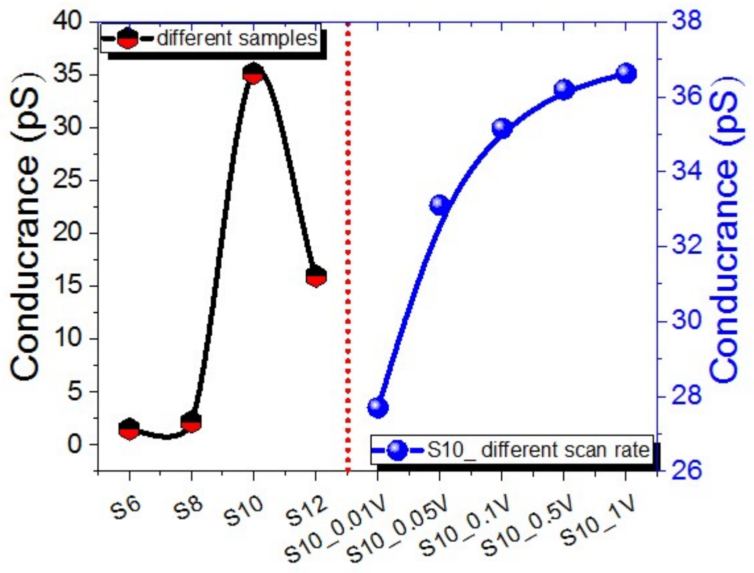

Figure S2. (A) electric conductance for the **Sn** series and **S10** sample at different step scans.

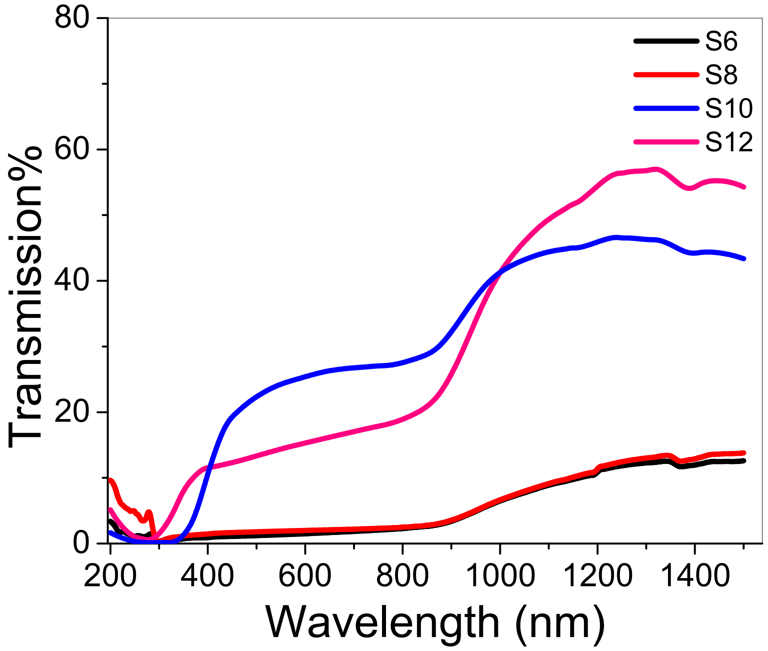

Figure S3.Optical transmittance of **Sn** films.

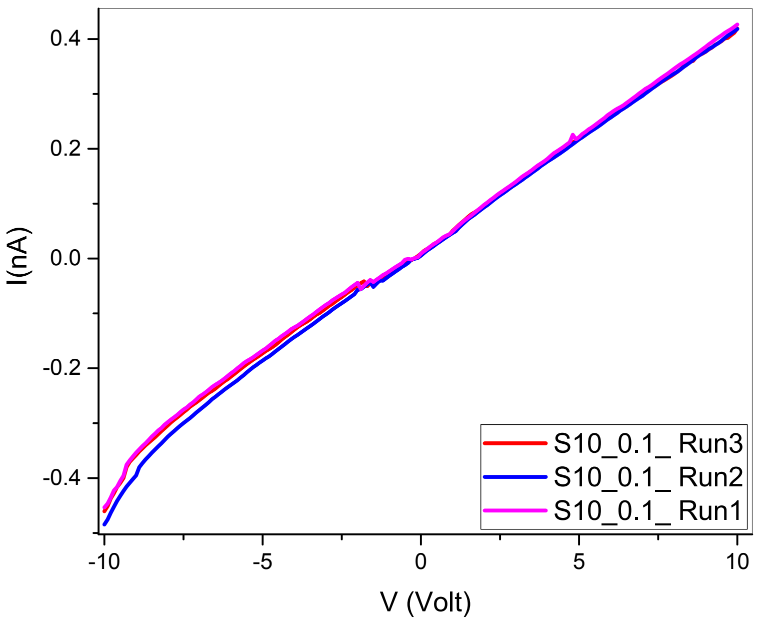

Figure S4. Current-Voltage characteristics of **S10** for different runs.
